# Supplementary material for: The Electrochemical Behavior of Unmodified and Pd-NPs Modified AB5 Hydrogen Storage Alloy in Selected Protic and Aprotic Ionic Liquids (ILs): Towards ILs-Based Electrolytes for Ni-MH Batteries
Source: Molecules. 2023 Jan 14;28(2):856. doi: 10.3390/molecules28020856 (PMC9865131; doi:10.3390/molecules28020856)
Supplement: Supplementary file 1 [file molecules-28-00856-s001.zip › molecules-2060241-supplementary.pdf]

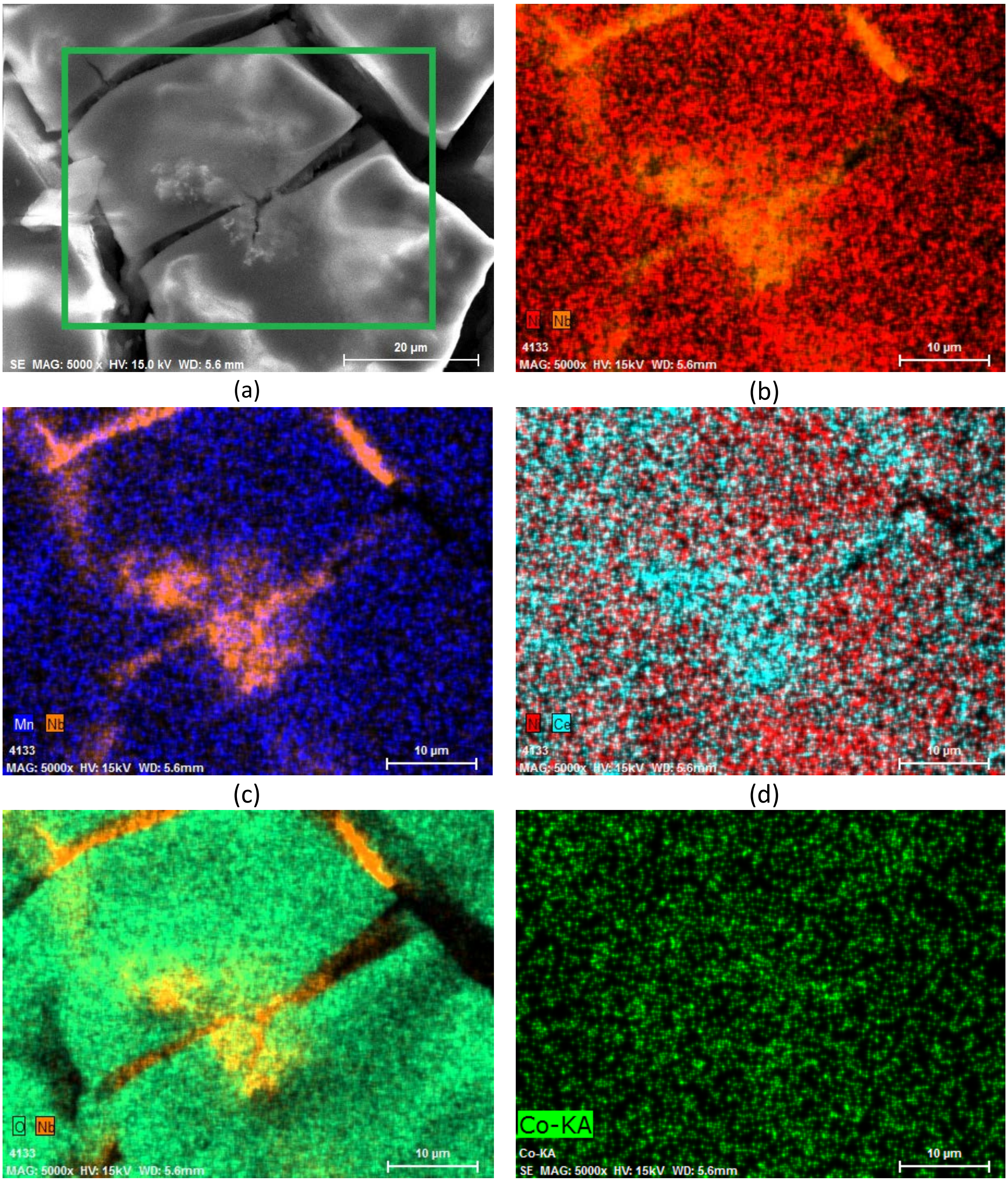
SE MAG: 5000 x HV: 15.0 kV WD: 5.6 mm
20 µm
Ni Nb
4133
MAG: 5000x HV: 15kV WD: 5.6mm
10 µm
Mn Nb
4133
MAG: 5000x HV: 15kV WD: 5.6mm
10 µm
Ni Ce
4133
MAG: 5000x HV: 15kV WD: 5.6mm
10 µm
O Nb
4133
MAG: 5000x HV: 15kV WD: 5.6mm
10 µm
Co-KA
Co-KA
SE MAG: 5000x HV: 15kV WD: 5.6mm
10 µm

(a) (b)

(c) (d)

(e) (f)

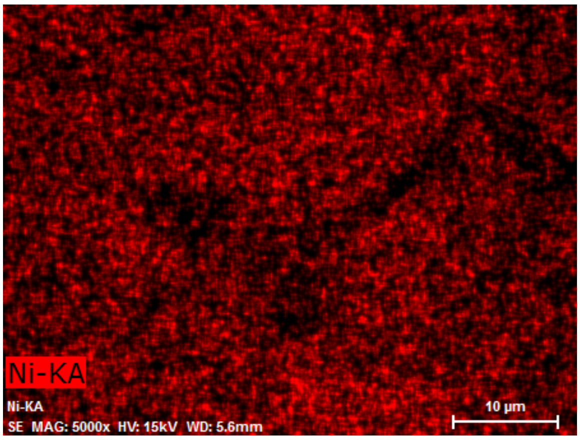
Ni-KA
Ni-KA
SE MAG: 5000x HV: 15kV WD: 5.6mm
10 µm
(g)

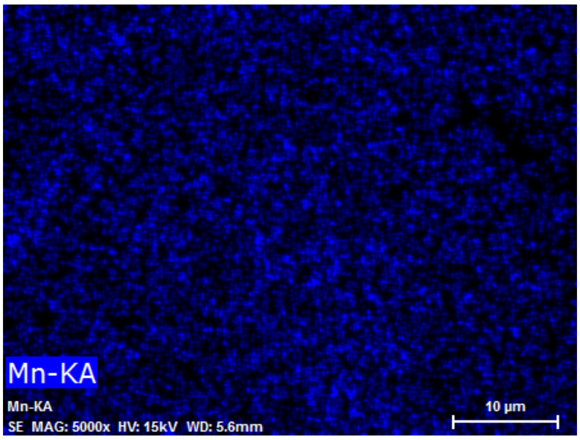
Mn-KA
Mn-KA
SE MAG: 5000x HV: 15kV WD: 5.6mm
10 µm
(h)

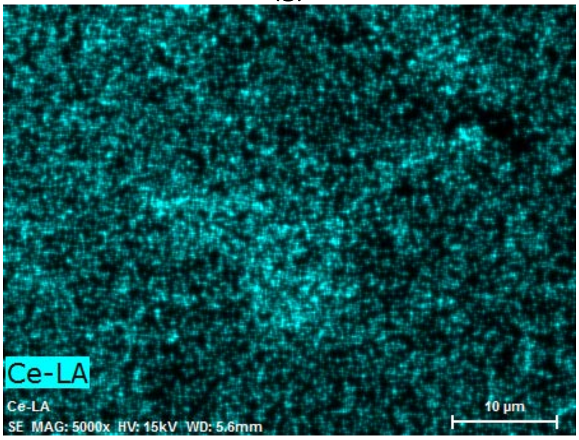
Ce-LA
Ce-LA
SE MAG: 5000x HV: 15kV WD: 5.6mm
10 µm
(i)

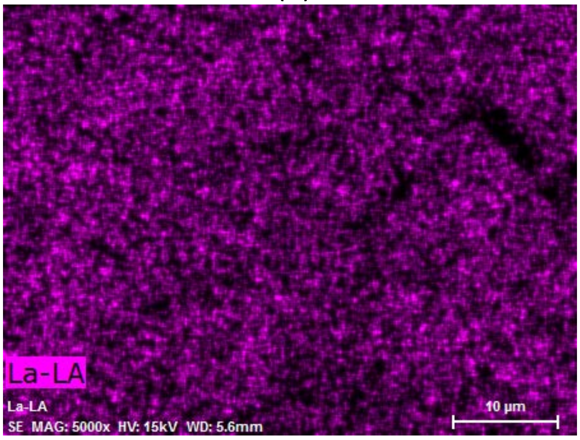
La-LA
La-LA
SE MAG: 5000x HV: 15kV WD: 5.6mm
10 µm
(j)

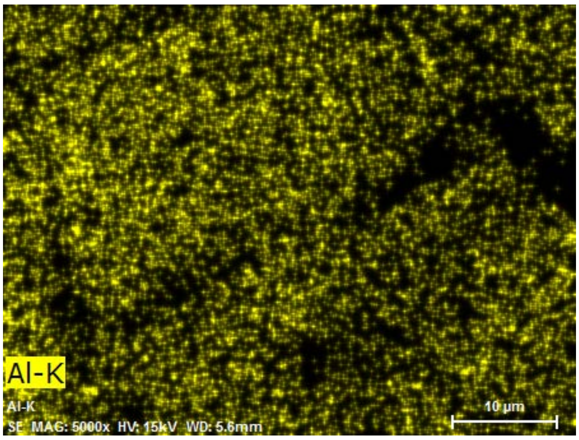
Al-K
Al-K
SE MAG: 5000x HV: 15kV WD: 5.6mm
10 µm
(k)

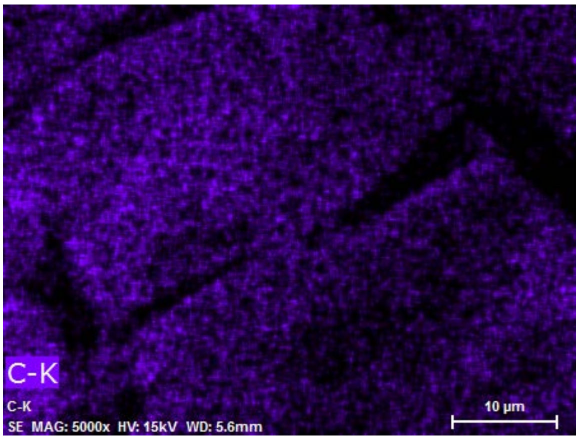
C-K
C-K
SE MAG: 5000x HV: 15kV WD: 5.6mm
10 µm
(l)

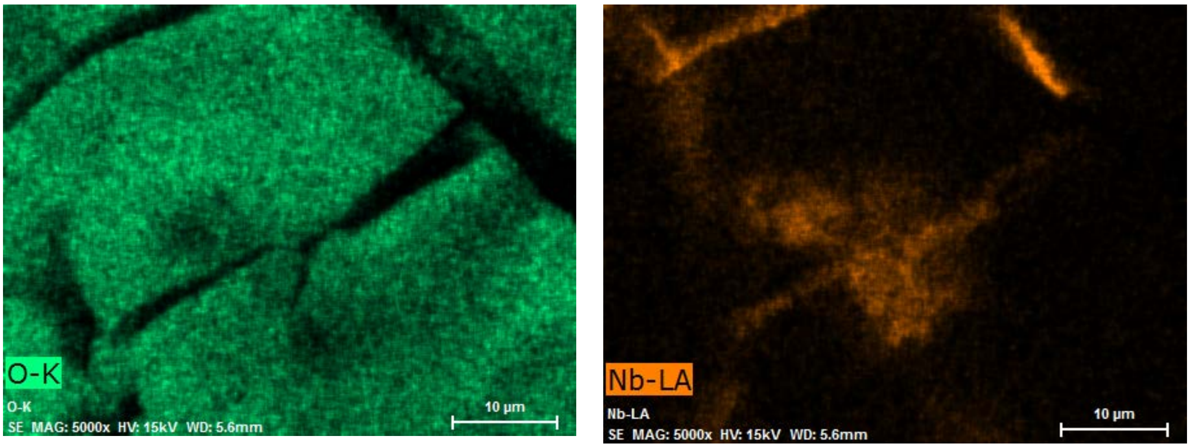

(m) (n)

Figure S1. *AB_5 alloy electrode after electrochemical treatment in 1 M HMS/EMIm-MS (a) SEM image – EDS mapping for the area marked with a green rectangle; EDS map of (b) Ni (red) and Nb (orange) (c) Mn (blue) and Nb (orange) (d) Ni (red) and Ce (cyan) (e) O (light green) and Nb (orange) (f) Co (green) (g) Ni (red) (h) Mn (blue) (i) Ce (cyan) (j) La (magenta) (k) Al (yellow) (l) C (violet) (m) O (light green) (n) Nb (orange)*
